# Web services-based text-mining demonstrates broad impacts for interoperability and process simplification

**DOI:** 10.1093/database/bau050

**Published:** 2014-06-10

**Authors:** Thomas C. Wiegers, Allan Peter Davis, Carolyn J. Mattingly

**Affiliations:** Department of Biological Sciences, North Carolina State University, 139 David Clark Lab, Campus Box 7617, Raleigh, NC 27695-7617, USA

## Abstract

The Critical Assessment of Information Extraction systems in Biology (BioCreAtIvE) challenge evaluation tasks collectively represent a community-wide effort to evaluate a variety of text-mining and information extraction systems applied to the biological domain. The BioCreative IV Workshop included five independent subject areas, including Track 3, which focused on named-entity recognition (NER) for the Comparative Toxicogenomics Database (CTD; http://ctdbase.org). Previously, CTD had organized document ranking and NER-related tasks for the BioCreative Workshop 2012; a key finding of that effort was that interoperability and integration complexity were major impediments to the direct application of the systems to CTD's text-mining pipeline. This underscored a prevailing problem with software integration efforts. Major interoperability-related issues included lack of process modularity, operating system incompatibility, tool configuration complexity and lack of standardization of high-level inter-process communications. One approach to potentially mitigate interoperability and general integration issues is the use of Web services to abstract implementation details; rather than integrating NER tools directly, HTTP-based calls from CTD's asynchronous, batch-oriented text-mining pipeline could be made to remote NER Web services for recognition of specific biological terms using BioC (an emerging family of XML formats) for inter-process communications. To test this concept, participating groups developed Representational State Transfer /BioC-compliant Web services tailored to CTD's NER requirements. Participants were provided with a comprehensive set of training materials. CTD evaluated results obtained from the remote Web service-based URLs against a test data set of 510 manually curated scientific articles. Twelve groups participated in the challenge. Recall, precision, balanced F-scores and response times were calculated. Top balanced F-scores for gene, chemical and disease NER were 61, 74 and 51%, respectively. Response times ranged from fractions-of-a-second to over a minute per article. We present a description of the challenge and summary of results, demonstrating how curation groups can effectively use interoperable NER technologies to simplify text-mining pipeline implementation.

**Database URL:** http://ctdbase.org/

## Introduction

The Comparative Toxicogenomic Database (CTD; http://ctdbase.org) is a publicly available, manually curated resource that promotes understanding of the mechanisms by which drugs and environmental chemicals influence biological processes and human health (1). CTD's PhD-level staff biocurators review the scientific literature and manually curate chemical–gene/protein interactions, chemical– disease relationships and gene–disease relationships, using a novel, highly structured notation in conjunction with CTD's Web-based curation tool (2). The manual curation process organizes disparate data from scientific publications into a standard structured format, making it more manageable and computable for bioinformatics-related processing. Curated data are integrated with each other as well as external data sets to facilitate development of novel hypotheses about chemical–gene–disease networks (1).

Curated data are captured using publicly available controlled vocabularies. Diseases are represented using CTD’s disease vocabulary, MEDIC (3), which merges OMIM (4) terms with the *Disease* subset of the National Library of Medicine’s Medical Subject Headings (MeSH) vocabulary (5), genes/proteins are represented using Entrez Gene terms (6), chemicals/drugs are represented using a modified subset of *Chemicals and Drugs* terms within MeSH (5) and chemical–gene/protein interactions are captured using CTD’s action term vocabulary (1).

CTD typically selects curation topics by targeting specific chemicals from a ‘Chemical Priority Matrix’ (2). Depending on the chemical targeted, there are often many more articles available for curation than can be reasonably processed by CTD biocurators. For example, a recent query for the chemical ‘arsenic’ at the PubMed interface from the National Center for Biotechnology Information (NCBI) Web site yielded >20 000 scientific articles. This is a common problem for biocurators. Consequently, text-mining is becoming an important component in the curation pipeline for the retrieval and extraction of biological information at curated databases (7), and has been shown to improve both the efficiency and accuracy of manual curation (8). WormBase (9), for example, has effectively leveraged machine learning methods to categorize the literature (10).

To ensure that CTD biocurators review only those articles that are most likely to yield curatable information within the context of CTD's structured curation paradigm, staff assessed the feasibility and potential advantages of implementing a text-mining pipeline (11). Based on the results of this study, CTD staff designed, developed, documented and implemented a highly effective, fully functional text-mining pipeline (12).

At the heart of the CTD text-mining pipeline is an internally developed, rules-based ranking algorithm that scores each article with a document relevancy score (DRS). Integral to the ranking algorithm is a set of locally installed, third party NER tools adapted for CTD use: Abner (13) for gene NER, Oscar3 (14, 15) for chemical NER and MetaMap (16) for disease recognition, as well as supplementary chemical and gene recognition. The effective deployment of these NER tools is essential to the success of DRS-based scoring, in that the algorithm scores articles based, in part, on the pervasiveness and spatial orientation of CTD’s controlled vocabulary terms in the text of an article’s title and abstract.

CTD is constantly exploring new ways to improve the effectiveness of DRS scoring. The BioCreative Workshop 2012 Track I/Triage track focused on document triaging for CTD (17). More specifically, participants developed tools that ranked articles in terms of their curatability, and identified gene/protein, chemical/drug and disease actors, as well as CTD interaction-related action terms. Top recall scores for gene, disease and chemical NER were 49, 65 and 82%. Although the results were impressive, they were of limited direct benefit to CTD because the NER tools were written using a wide variety of technologies and within technical infrastructures and architectures that would not necessarily easily integrate directly into CTD's existing text-mining pipeline. In short, interoperability and integration complexity were major impediments to the direct application of the NER-related aspects of the collaboration to the CTD pipeline. Impediments included lack of NER process modularity, operating system and programming language incompatibility, tool configuration complexity, lack of standardization of high-level inter-process communications and database management system-related incompatibility.

**Figure 1.**
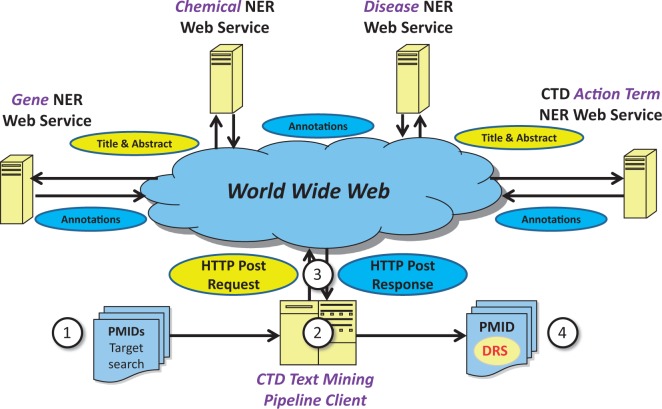
Web service-based NER logical design. Under a Web service-based conceptual design, (1) a list of potentially relevant PubMed IDs (PMIDs) is secured via a search of PubMed, typically for a target chemical. (2) The list is processed asynchronously by batch-oriented processes. Rather than performing NER using locally installed NER tools, (3) HTTP calls containing text passages are made to remote Web services; the results of NER are used as a key component in document ranking algorithms. (4) PMIDs are then assigned a DRS by the document ranking algorithms.

One approach to potentially mitigate NER-related interoperability and general integration issues is the use of Web services for NER. Web services are designed to accommodate interoperable machine-to-machine interaction over the Web (18). Rather than locally installing NER tools directly into the CTD asynchronous, batch-oriented text-mining pipeline, Web services provide the capability for CTD’s text-mining pipeline to make simple HTTP-based calls to remote NER Web services for gene/protein, chemical/drug, disease and/or action term recognition (Figure 1). This approach tends to be inherently simpler than direct local pipeline integration because the technical details of the tools themselves are completely abstracted by the Web service. Alternatively, the problems imposed by local tool installation are many, ongoing and nontrivial. Local tool installation and integration requires text-mining pipeline developers to address tool-specific issues such as operating system compatibility, programming language interpretation/compilation-related environments, tool versioning maintenance and control, tool-associated library compatibility, tool configuration maintenance, process modularity, inter-process communications and multi-thread capacity. The potential benefits of implementing a Web serviced-based approach to NER for CTD and other Web-based resources from a conceptual perspective were sufficient to use Track 3 as a mechanism to further study this approach.

The design of the track was predicated on one essential requirement––although internally the Web Services could be radically different from one another, externally all sites should behave identically from a communications perspective and be completely interchangeable. It was therefore critical that sites use one standard form of high-level inter-process communications. As Track 3 tasks were being analyzed and designed by CTD staff, a group of NCBI-led collaborators were concurrently and coincidentally working on the development of BioC, a common interchange format to represent, store and exchange data in a simple manner between different language processing systems and text-mining tools (19). The more CTD learned about and participated in development of BioC, the more it became clear that BioC's simple, lightweight, flexible design, along with its planned support across multiple programming languages and operating environments, made it an extremely attractive vehicle for Track 3 high-level inter-process communications.

The CTD track of BioCreative IV focused on NER interoperability and tool complexity abstraction. Participants were asked to build interoperable, Web service-based tools that would enable CTD to send text passages to their remote sites to identify gene/protein, chemical/drug, disease and chemical/gene-specific action term mentions, each within the context of CTD’s controlled vocabulary structure, using BioC for inter-process communications. The challenge was to determine whether teams such as CTD could benefit from text-mining tools accessed remotely and developed using a common interoperable communications framework. And, if so, would the response time associated with such tools be suitable for asynchronous, batch processing-based text-mining using technologies such as Web services?

## Methods and materials

### Web service architectural style

Representational State Transfer (REST) was selected as the architectural style for the participant Web services. REST was originally designed to abstract the architectural elements of distributed systems by enabling client processes to ignore the details of component implementation and protocol syntax to instead focus on the role of the components, enhancing simplicity by providing a clean separation of concerns, and hiding the underlying implementation of resources and communication mechanisms (20). The primary purpose of REST-compliant Web services is to manipulate XML representations using a uniform set of stateless operations (21); the term stateless in the Web service context means that requests are processed without the knowledge of any prior requests. The stateless nature of REST tends to improve scalability because the Web service need not store state information between requests, allowing the server component to quickly free resources; moreover, the stateless feature simplifies implementation because the server need not manage resource usage across requests (20). Although other Web service-based options were available, the timely emergence of BioC, coupled with REST's XML-centric nature and other attractive design features, made a REST/BioC-compliant architecture well positioned for Track 3 use.

### Training phase

**Figure 2.**
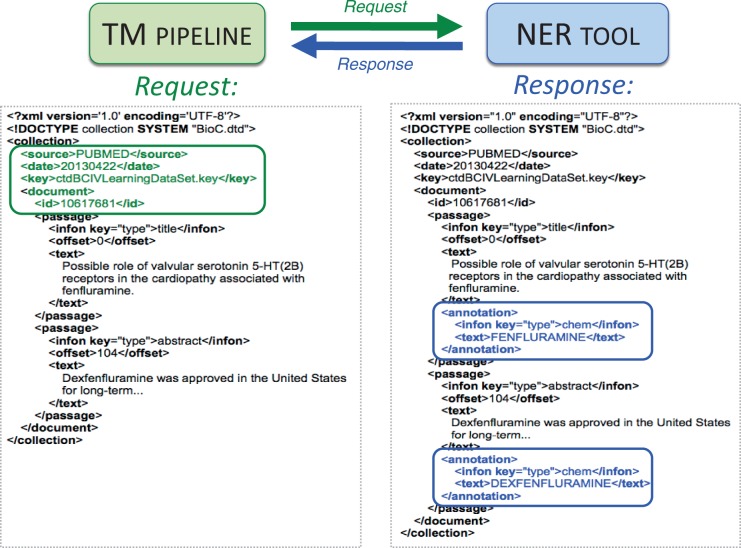
BioC-based high-level inter-process communications. A sample request in BioC format is sent by Web service from the text-mining (TM) pipeline to the NER tool (green arrow). The PubMed ID, title, abstract and designated key file describing the semantics of the data are included within the XML request (left, green box). A chemical-specific response is returned from the NER tool to the TM pipeline (blue arrow). The NER Web service reads the BioC XML and attempts to identify chemicals in the title and abstract. Here, two chemical entities (fenfluramine and dexfenfluramine) are identified as BioC annotation objects for the NER chemical category in the response (right, blue boxes).

For participants to gain an understanding of CTD curation and associated NER requirements, participants were provided with a comprehensive set of training materials in May 2013. A detailed document, entitled *Summary Of Curation Details For The Comparative Toxicogenomics Database*, was distributed to participants (http://ctdbase.org/reports/CTD_curation_summary.docx). In addition, a training data set was made available that consisted of 1112 articles previously manually curated by CTD biocurators, and included 3511 distinct curated genes, 3144 chemicals, 1965 diseases and 2521 chemical/gene-specific action terms, all within the context of 9877 manually curated interactions (Table 1). The training data set was provided in a single BioC XML-based file, and contained important details associated with the articles in the data set, including the PubMed ID, title, abstract, gene, chemical, disease and action term annotations, and a list of associated curated interactions. References to general BioC information, BioC DTDs and a key file that described the BioC XML format in the context of the training data set, were also provided to participants, as were sample Web service requests and responses for each NER category (Figure 2). Finally, the complete CTD controlled vocabularies, in multiple formats and including both terms and synonyms, were provided for each of the NER categories.

**Table 1.** Training and test data sets

	Training data set	Test data set
Total scientific articles	1112	510
Distinct genes/proteins	3511	1122
Chemicals/drugs	3144	1192
Diseases	1965	943
Action terms	2521	966
Interactions	9877	3953

An overview is provided of the training and test data sets.

**Figure 3.**
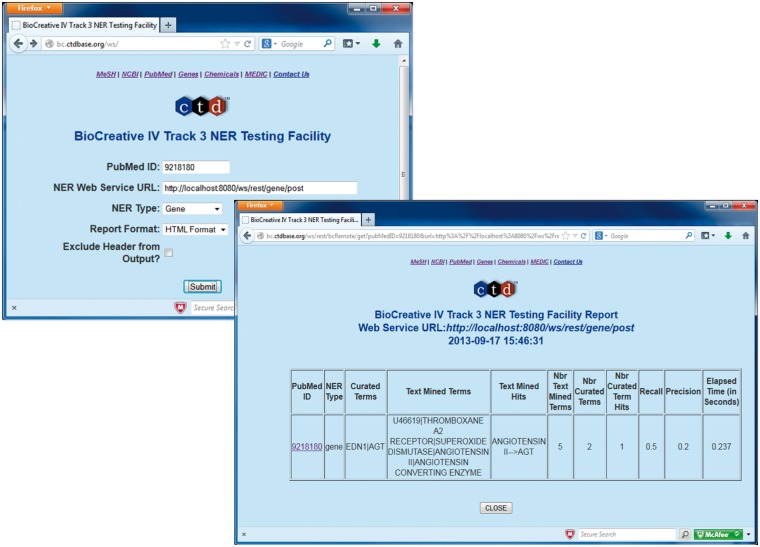
BioCreative IV Track 3 NER Testing Facility. Participants were provided with the *BioCreative IV Track 3 NER Testing Facility* developed by CTD. This testing facility provided a front-end to a CTD Web service that on execution called the participant's Web service using BioC XML associated with a specified PubMed ID for inter-process communications (top left screenshot). CTD's Web service would in turn receive text-mined annotations from the participant's Web service (using BioC XML). CTD's Web service then processed the annotations and computed the results against the curated data set, providing the user with recall, precision, response time and a detailed list of curated terms, text-mined terms and text-mined term hits (bottom right screenshot).

In July 2013, the BioCreative IV, Track 3 NER Testing Facility Web site was released (Figure 3). This testing facility provided a front-end to a CTD Web service that, on execution, called the participant's Web service, enabling participants to test their Web services against the training data set. More specifically, the participants simply entered a PubMed ID (from the training data set), the URL of their Web service, an NER type (i.e. gene, chemical, disease or action term) and report format-related information. This would cause CTD's Web service to call the participants Web service using BioC XML (associated with the specified PubMed ID) for inter-process communications; CTD's Web service would in turn receive text-mined annotations from the participant's Web service using BioC XML (Figures 1 and 2). CTD's Web service would then process the annotations and compute the results against the curated data set, providing the user with recall, precision, response time and a detailed list of curated terms, text-mined terms and text-mined term hits (i.e. curated terms successfully identified by the text-mining tools—either by a synonym to the term or by the term itself). The participants were also given the opportunity to bypass the Web-based front-end and call the CTD Web service directly via application-to-application HTTP GET calls; this feature enabled users to run batch processes against the entire training data set. The testing facility was heavily used, receiving >260 000 hits from its inception through completion of the training phase.

### Testing phase and methodology

On 19 August 2013, participants were asked to submit to Track 3 organizers Web service URLs for testing, as well as brief system descriptions. CTD staff then tested the Web services against a test data set of 510 articles manually curated by CTD staff, using a client process specifically developed for testing. The process tested one abstract at a time, and participants were unaware of the articles to be tested before testing. The test data set included 1122 distinct curated genes, 1192 chemicals, 943 diseases and 966 chemical/gene-specific action terms, all within the context of 3953 manually curated interactions (Table 1).

Recall, precision, balanced F-score (sometimes referred to as F1 score or F-measure) and response times were captured for each Web service call. Recall scores were calculated by dividing the number of distinct curated actors identified by the text-mining tools—either by a synonym to the term or by the term itself—by the total number of distinct curated actors. Precision scores were calculated by dividing the number of distinct curated actors identified by the text-mining tools by the number of distinct text-mined terms. Balanced F-scores were calculated as follows:

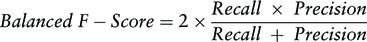



Balanced, rather than weighted, F-scores were calculated because CTD equally values recall and precision. Since NER tools are currently used to rank documents at CTD, it is important to maximize recall while at the same time minimizing false positives. Other groups may have different priorities and therefore weight, calculate and evaluate metrics differently.

Response times were calculated by measuring the duration between the call from CTD to the respective Web service, and receipt of communications back to CTD from the Web service. All testing was performed by CTD’s software engineer, and was completed at the Mount Desert Island Biological Laboratory in Bar Harbor, Maine, USA. Micro-averaging was used for aggregate recall, precision, F-score and response time.

### Limitations

It is important to note that the standard text-mining metric calculations of precision and recall may be imperfect within the context of CTD curation. The gold standard data were composed of curated—rather than mentioned—gene/protein, disease and chemical/drug actors within each abstract. There are likely to be instances where valid actors are not actually involved in the types of interactions captured by CTD curators; furthermore, there are also likely instances where curated actors are identified by curators only in the full text of the article. Consequently, the complete universe of valid actor mentions specifically resident within each title and abstract is not recorded by CTD curators, and is therefore unknown. In addition, the location of the curated actors within the title and abstracts is not tracked by CTD curators; consequently, the specific location of the actors identified by the NER tools was not tested.

## Results and discussion

Twelve groups participated (Table 2), submitting a combined total of 44 Web services for testing. Of the 44 Web services submitted, 39 were successfully tested against the complete test data set, including 9 gene-, 10 chemical-, 10 disease- and 10 action term-based NER Web services. The remaining five Web services were fully operational, but were unable to process the complete test data set for varying reasons. In three of the five cases the vast majority of PubMed articles in the data set were successfully processed; however, it appeared as though preprocessing/indexing of the individual PubMed abstracts was necessary before actual submission, and some of the abstracts in the test data set had not yet been preprocessed. The reasons for failure of the remaining Web services were unclear.

**Table 2.** Participating teams

Institution/department	NER tool summary	Primary contact <email address>	Web service URL(s)
National ICT Australia/Victoria Research Laboratory (Melbourne, Australia)	Gene, chemical, disease and action term: dictionary-based lookup using ConceptMapper (22), with custom dictionary construction algorithms and parameter tuning.	Andrew MacKinlay <Andrew.MacKinlay@nicta.com.au>	To be determined
Wuhan University (Wuhan, Hubei, China)	Gene, chemical and disease: a proprietary dictionary-based NER tagger	Cheng Sun <whucsnlp@gmail.com>	http://nlp.whu.edu.cn/Bio_NER/gene
			http://nlp.whu.edu.cn/Bio_NER/chem
			http://nlp.whu.edu.cn/Bio_NER/disease
			http://nlp.whu.edu.cn/Bio_NER/action_term
	Action term: LIBSVN (23)		
University of Applied Sciences of Western Switzerland, Geneva/BiTeM Group, Information Science Department (Geneva, Switzerland)	Gene: NormaGene (24)	Dina Vishnyakova <dina.vishnyakova@unige.ch>	http://pingu.unige.ch:8080/Toxicat/rest/gene/post/
	Diseases and chemicals: *ad hoc* keyword recognizer based on the controlled vocabulary dictionaries provided by CTD		http://pingu.unige.ch:8080/Toxicat/rest/chem/post/
			http://pingu.unige.ch:8080/Toxicat/rest/disease/post/
			http://pingu.unige.ch:8080/Toxicat/rest/action_term/post/
	Action Terms: GOCat (25), a proprietary thesaurus and machine learning-based system		
University and University Hospitals of Geneva/Division of Medical Information Sciences (Geneva, Switzerland)			
SIB Swiss Institute of Bioinformatics/SIBtex (Geneva, Switzerland)			
University of Zurich/Institute of Computational Linguistics (Zurich, Switzerland)	Gene, chemical and disease: Ontogene (26), a lexical lookup and machine learning-based entity tagger.	Fabio Rinaldi <fabio.rinaldi@uzh.ch>	http://kitt.cl.uzh.ch:8081/gene
			http://kitt.cl.uzh.ch:8081/chem
			http://kitt.cl.uzh.ch:8081/disease
			http://kitt.cl.uzh.ch:8081/action_term
	Action terms: a document classifier-based entity tagger		
Academia Sinica/Institute of Information Science (Taipei, Taiwan)	Gene: BioC-GN, a proprietary machine learning- and dictionary-based NER module.	Hong-Jie Dai <hjdai@tmu.edu.tw>	http://bws.iis.sinica.edu.tw/BioC_GN/RestServiceImpl.svc/gene
Yuan Ze University/Department of Computer Science & Engineering (Taoyuan, Taiwan)			
Taipei Medical University/Graduate Institute of BioMedical Informatics (Taipei, Taiwan)			
National Tsing-Hua University/Department of Computer Science (HsinChu, Taiwan)			
National Central University/Department of Computer Science and Information Engineering (Zhongli City, Taiwan)
National Cheng Kung University/Department of Computer Science and Information Engineering (Tainan, Taiwan)	Gene, chemical, disease and action term: adapted version of CoINNER, a proprietary dictionary/conditional random fields-based NER tagger	Hung-Yu Kao <hykao@mail.ncku.edu.tw>	http://140.116.245.192:8080/coinner/gene
			http://140.116.245.192:8080/coinner/chemical
			http://140.116.245.192:8080/coinner/disease
			http://140.116.245.192:8080/coinner/action_term
National Cheng Kung University/Department of Computer Science and Information Engineering (Tainan, Taiwan)	Gene, chemical, disease and action term: adapted version of GCDA, a proprietary dictionary- and search engine-based NER tool that integrates OSCAR4 (27), LingPipe (28), MetaMap (16) and Lucene (29)	Jiun-Huang Ju <jujh@iir.csie.ncku.edu.tw>	http://rose.csie.ncku.edu.tw:8080/rest/gene
		Jung-Hsien Chiang <jchiang@mail.ncku.edu.tw>	http://rose.csie.ncku.edu.tw:8080/rest/chem
			http://rose.csie.ncku.edu.tw:8080/rest/disease
			http://rose.csie.ncku.edu.tw:8080/rest/action_term
Mayo Clinic/Department of Health Sciences Research (Rochester, MN, USA)	Gene, chemical and disease: a proprietary dictionary-based and conditional random fields-based NER tagger, integrating BioTagger-GM (30), OSCAR4 (27), Chemspot (31), GENIA POS (32) and Mallet (33)	Komandur Elayavilli, Ravikumar <KomandurElayavilli.Ravikumar@mayo.edu>	http://50.63.53.51:8085/gene
			http://50.63.53.51:8085/chemical
			http://50.63.53.51:8085/disease
OntoChem GmbH (Halle/Saale, Germany)	Gene, chemical, disease, and action term: adapted version of OCMiner (34), a proprietary dictionary-based text mining pipeline	Matthias Irmer <matthias.irmer@ontochem.com>	http://webservice.ontochem.com:49916/ocm/xml/gene/
			http://webservice.ontochem.com:49916/ocm/xml/chem/
			http://webservice.ontochem.com:49916/ocm/xml/disease/
			http://webservice.ontochem.com:49916/ocm/xml/action_term/
University of Manchester/National Centre for Text Mining (Manchester, UK)	Gene, chemical and disease: a conditional random fields model built with NERSuite (35) in conjunction with additional syntactic and semantic features.	Rafal Rak <rafal.rak@manchester.ac.uk>	http://nactem.ac.uk/CTDWebService/ctd/gene
			http://nactem.ac.uk/CTDWebService/ctd/chem
			http://nactem.ac.uk/CTDWebService/ctd/disease
			http://nactem.ac.uk/CTDWebService/ctd/action_term
	Action term: Support Vector Machine-based model with dictionary- and co-occurrence-based features.		
RelAgent Technologies Pvt. Ltd. (Adyar, Chennai, India)	Gene, chemical, disease and action term: adapted version of Cocoa, a proprietary dictionary/rule based entity tagger	S V Ramanan <ramanan@relagent.com>	http://npjoint.com/Cocoa/api/ctd/gene/
			http://npjoint.com/Cocoa/api/ctd/chem/
			http://npjoint.com/Cocoa/api/ctd/disease/
			http://npjoint.com/Cocoa/api/ctd/action_term/
Anna University/AU-KBC Research Centre (Chrompet, Chennai, India)	Gene, chemical, disease and action term: a proprietary CRF++ (36) based NER tagger	Sindhuja Gopalan<sindhujagopalan@au-kbc.org>	http://124.124.220.114/Jersey/rest/gene/post
		Sobha Lalitha Devi<sobha@au-kbc.org>	http://124.124.220.114/Jersey/rest/chem/post
			http://124.124.220.114/Jersey/rest/disease/post
			http://124.124.220.114/Jersey/rest/action_term/post

Twelve teams participated in BioCreative IV, Track 3, submitting a combined total of 44 Web services for testing. Although all of the Web services were fully operational, five of the services could not process the entire Track 3 test data set. The participating institutions, brief descriptions of the basic design of the tools, along with the associated Web service URLs and points-of-contact, are provided. Note that in most cases the four NER categories (i.e. gene, chem, disease and action term) are integral to the URL naming nomenclature. Also, be aware that the majority of the URLs provided are machine-to-machine URLs, and have no associated graphical user interfaces.

### Gene/protein NER results

**Figure 4.**
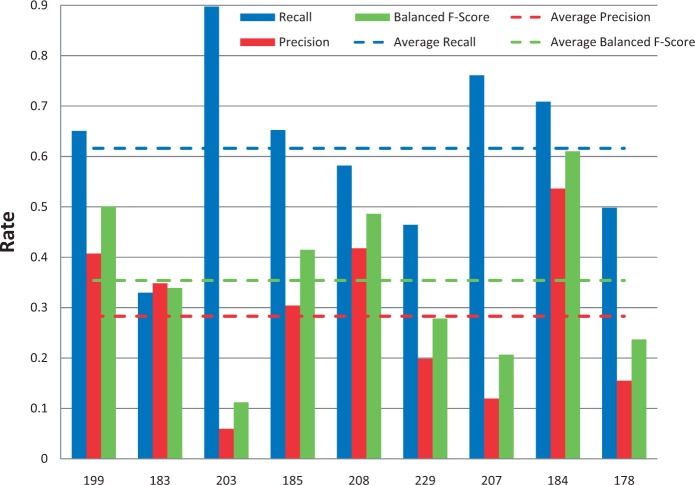
Gene/protein named-entity recognition. Gene recall (blue), precision (red) and balanced F-score (green) results are shown for each participating group (anonymously identified by group number on x-axis). Average scores for each metric (dotted lines) are also provided.

**Figure 5.**
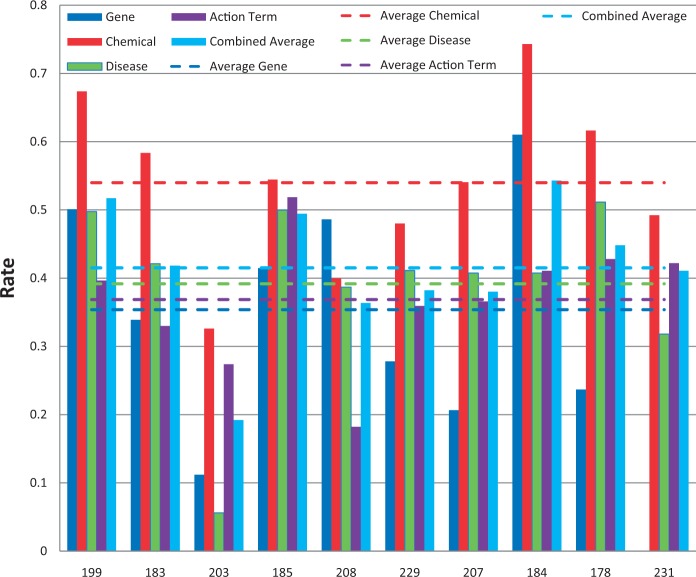
Balanced F-scores by group. Balanced F-score results for each NER category, as well as a combined average, are provided for each participating group (anonymously identified by group number on x-axis). Average scores for each metric (dotted lines) are also provided.

**Figure 6.**
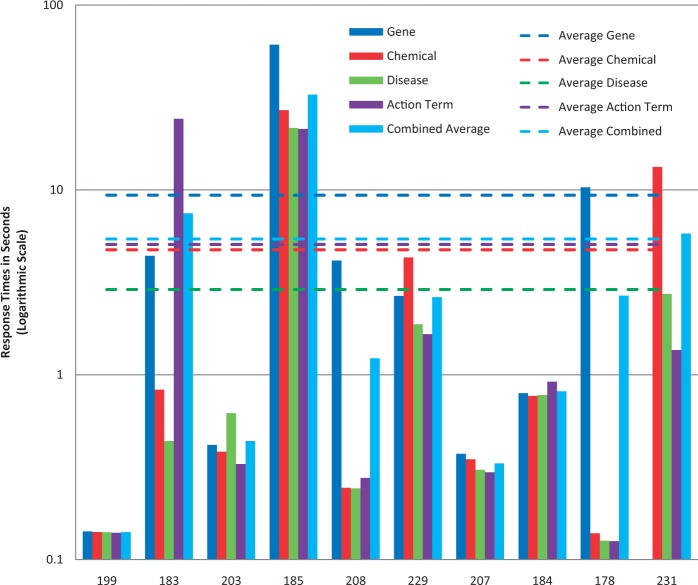
Response times. Response time results for each NER category, as well as a combined average, are provided for each participating group (anonymously identified by group number on x-axis). *Note*: the response time in seconds (y-axis) uses a logarithmic scale.

Among the 12 submissions for gene/protein NER, nine were successfully tested. As shown in Figure 4, average recall was 62% and ranged from 32 to 89%. Average precision was 28% and ranged from 6 to 54%. Average balanced F-scores were 36% and ranged from 11 to 61% (Figures 4 and 5). Interestingly, the two groups with the highest recall scores also had the lowest precision and F-scores, suggesting a much stronger emphasis placed on recall at the expense of precision. The average response time was 9.4 s and ranged from 0.14 to 61 s, with a large standard deviation of 19.6 s (Figure 6).

### Chemical/drug NER results

**Figure 7.**
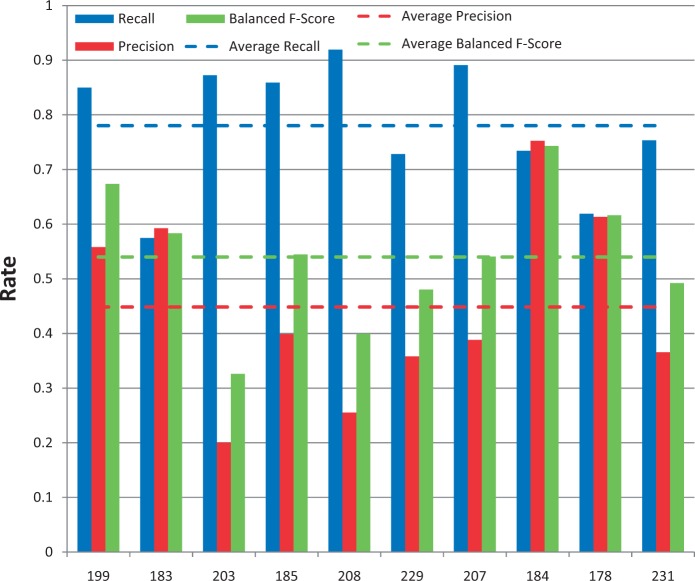
Chemical/drug named-entity recognition. Chemical recall (blue), precision (red) and balanced F-score (green) results are shown for each participating group (anonymously identified by group number on x-axis). Average scores for each metric (dotted lines) are also provided.

Among the 11 submissions for chemical/drug NER, 10 were successfully tested (Figure 7). Average recall was 78% and ranged from 57 to 92%. Average precision was 45% and ranged from 20 to 75%. Average balanced F-scores were 54% and ranged from 33 to 74% (Figures 5 and 7). The average response time was 4.7 s and ranged from 0.14 to 27 s, with a standard deviation of 8.8 s (Figure 6).

### Disease NER results

**Figure 8.**
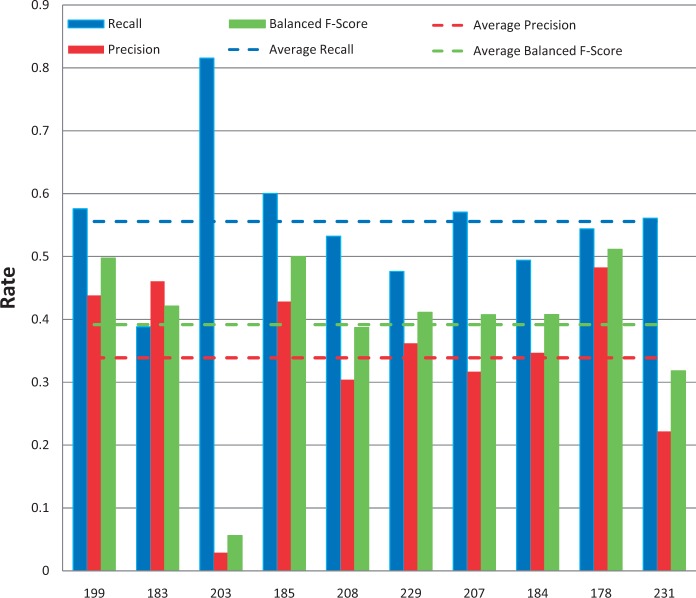
Disease named-entity recognition. Disease recall (blue), precision (red) and balanced F-score (green) results are shown for each participating group (anonymously identified by group number on x-axis). Average scores for each metric (dotted lines) are also provided.

Among the 11 submissions for disease NER, 10 were successfully tested (Figure 8). Average recall was 56% and ranged from 39 to 82%. Average precision was 34% and ranged from 3 to 48%. Average balanced F-scores were 39% and ranged from 6 to 51% (Figures 5 and 8). The average response time was 2.9 s and ranged from 0.13 to 22 s, with a standard deviation of 6.6 s (Figure 6).

### Chemical/gene action term NER results

**Figure 9.**
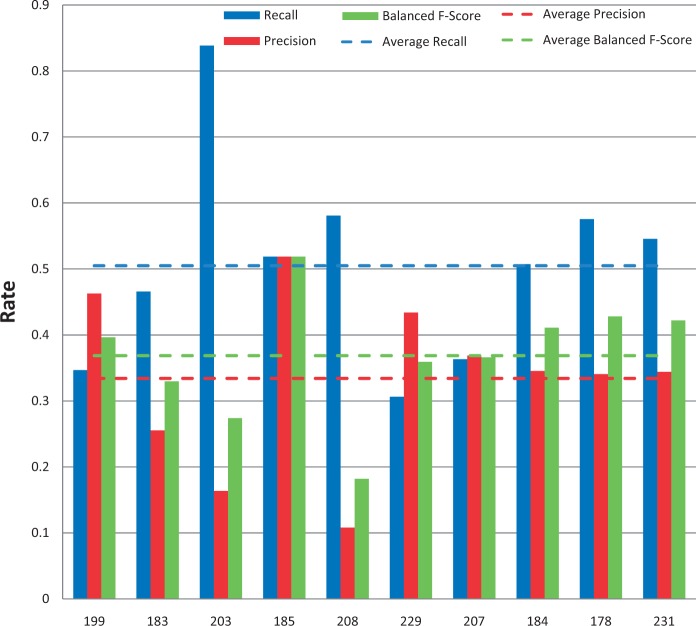
Action term named-entity recognition. Chemical/gene action term recall (blue), precision (red) and balanced F-score (green) results are shown for each participating group (anonymously identified by group number on x-axis). Average scores for each metric (dotted lines) are also provided.

All of the 10 submissions for chemical/gene action NER were successfully tested (Figure 9). Average recall was 50% and ranged from 31 to 84%. Average precision was 33% and ranged from 11 to 52%. Average balanced F-scores were 37% and ranged from 18 to 52% (Figures 5 and 9). The average response time was 5.1 s and ranged from 0.13 to 24 s, with a standard deviation of 9.4 s (Figure 6).

### Aggregate F-score results

A total of 10 groups submitted 39 Web services that were successfully tested. Average combined balanced F-score was 41% and ranged from 19 to 54% (Figure 5).

### Response time results

The combined average response time across NER categories was 5.4 s per article, with a standard deviation of 9.9 s (Figure 6). The fastest combined average response time was 0.14 s and the slowest was 32.8 s. The variability appeared to be a function of the NER process itself and/or the hardware on which the process was run. There was no apparent correlation either with geographic area or NER recall or precision performance.

### Combined results

**Figure 10.**
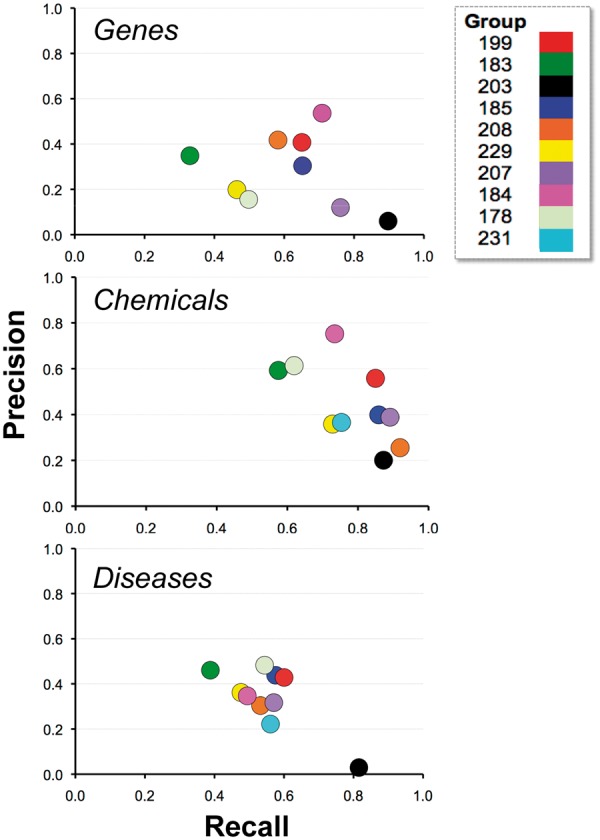
Recall and precision. Combined average recall (x-axis) and precision (y-axis) results are shown for each participating group (color-coded by group number) within major NER category. For some groups there appeared to be a clear trade-off between recall and precision (e.g. 203), whereas for other groups trade-offs were less apparent (e.g. 184 and 199).

**Figure 11.**
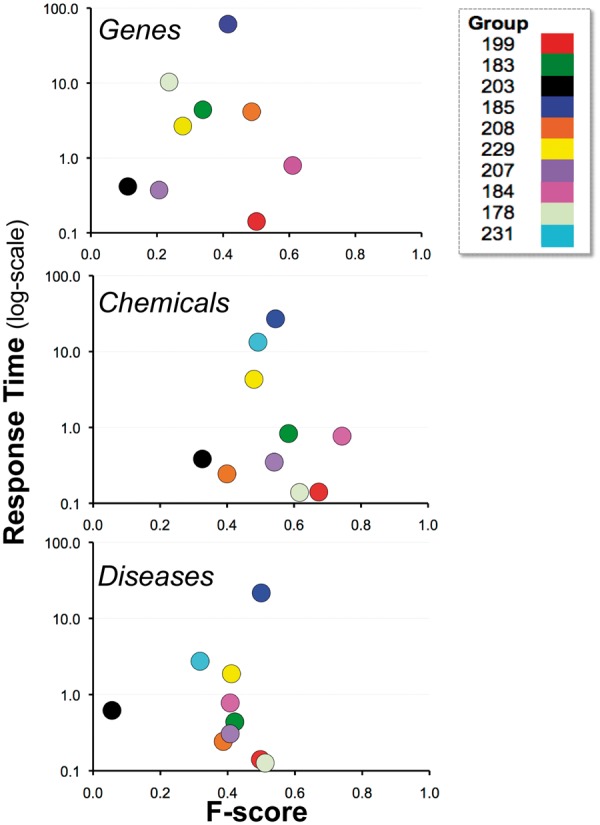
Balanced F-score and response time. Combined average balanced F-score (x-axis) and response time (y-axis) results are shown for each participating group (color-coded by group number) within major NER category. There was no clear relationship between response time and F-score. *Note*: the response time in seconds (y-axis) uses a logarithmic scale.

Figure 10 depicts the relationship between recall and precision, providing the combined results by group within NER category. For some groups there appeared to be a clear trade-off between recall and precision (e.g. 203), whereas for other groups, trade-offs were less apparent (e.g. 184 and 199). Note also that the gene and chemical categories were highly stratified, whereas the disease NER results were more tightly organized. Figure 11 similarly contrasts balanced F-score with combined average response time by group within NER category. There was no clear relationship between response time and F-score.

### Discussion

The results of Track 3 testing clearly validate the conceptual feasibility of integrating Web service-based NER functionality into asynchronous batch-oriented text-mining pipelines. The participant NER performance and Web service response times were more than adequate for such use in most cases. In making these assertions it must be emphasized that, within margins, equal weight was placed on response times as was placed on NER performance in evaluating Track 3. Although response times might seem secondary to NER performance, a limiting factor to remote processing of the type proposed here is clearly response time: if the best NER tools have inadequate response times, they are of little use to resources like CTD. At times the CTD text-mining pipeline processes tens of thousands of abstracts; poor response times can equate to days to process large data sets even in cases where multi-threading is introduced. For example, the worst gene NER response time was 1 min per article, which for a 6000 article data set equates to 100 h of sequential processing for gene NER alone, whereas a response time of 5 s equates to a much more manageable 8.3 h of sequential processing. CTD targets an average response time of ≤10 s for non–thread-safe tools.

Among the most positive findings of Track 3 is that neither geographic location nor NER performance appeared to play a role in response times; some of the best NER-performing groups had the fastest response times. Group 184, for example, delivered strong performance in every NER category with fraction-of-a-second response times. Group 199 delivered similar results with even faster response times, and some of the more remote groups provided among the fastest response times (specifics are not provided to maintain group anonymity). One of the group's response times was largely dependent on whether the article in question had been pre-processed; once the article had been indexed, the response time moved from in excess of 10 s down to fractions-of-a-second for the remaining major NER types. Even the group with the slowest response times, 185, delivered above average NER recall and precision performance and attributed their slow response times to inferior hardware rather than anything inherent to the NER process itself. From a conceptual perspective, the results were encouraging, providing a proof-of-concept that Web services-based NER is feasible for asynchronous processing.

Chemicals/drugs continue to be the strongest actor NER recall category on average (78%), mirroring past CTD-related studies (11, 12, 17), followed by genes (62%) and diseases (56%). One of the reasons disease NER may be more difficult than the other major vocabularies is because there might be more ways in which authors can express a disease term than is the case with the other terms. For example, a study of the CTD controlled vocabularies shows that there are on average many more synonyms for disease terms (6.4) than for genes/proteins (3.6) and chemicals/drugs (3.0). This trend suggests that there are more diverse ways to describe disease phenomena. As disease vocabularies become better defined, standardized and more commonly accepted (3), this trend might decrease, and the NER could increase to a comparable level to what is currently seen for genes and chemicals.

The performance of action terms NER was far superior to the BioCreative 2012 results (17), but continued to be somewhat disappointing; although the top recall score was 84%, the precision for that score was 16%, one of the lowest in the group. Because the population of potential values is small for action terms in relation to the other NER categories, greater emphasis is placed on precision scores, and the 33% average precision score was disappointing. One exception was Group 185, which scored >50% in both recall and precision. Action term recognition is an extremely important area for CTD in that the ability to accurately recognize these terms would significantly improve CTD's ability to identify articles that contain curatable information. More modeling, analysis and design work needs to be done by CTD, either internally or in conjunction with other collaborators, to develop NER algorithms and tools to better recognize actions terms when they appear in text.

The performance of several tools integral to Track 3 merit note. BioC proved to be an extremely robust, effective tool in standardizing high-level inter-process communications. The framework provided all the functionality required for Track 3, and did so in an unobtrusive fashion: the vast majority of the participants required little, if any, help from the organizers with respect to BioC, there were few syntax errors in the BioC XML returned from the Web services, and those syntax errors that did exist, for the most part, were quickly corrected. The fact that CTD did not have to create an application-specific inter-process communications framework was very beneficial to the track, and in the end the tools developed for the track provided a level of interoperability that would not have otherwise existed in the absence of BioC.

The REST-compliant architecture also proved to be an excellent design choice because its design goal to abstract the architectural elements of distributed systems (20) complemented the Track 3 goal. In addition, the fact that so many fully operational NER Web services were set up within a few months, communicating via a common framework, speaks to the usability of the REST architecture style.

The NER Testing Facility Web site was heavily used and successful in ensuring that the groups were producing Web services that were in compliance with the requirements established for Track 3. The fact that all 12 groups provided functional Web services (if not fully functional against the entire test data set for two of the groups) lends support to the effectiveness of the testing facility. The site provided a simple way to test applications during the training phase of the project to ensure the correct syntax. The feature that enabled users to bypass the Web-based front-end and call the CTD Web service directly via application-to-application HTTP GET calls provided participants with the ability to process and refine their NER performance against the entire training data set.

From a conceptual perspective, we would be remiss if we did not note that a Web service-based approach has shortcomings and limitations that should not be minimized, from both the server side and the client side. From the server side, the implementation of Web services clearly presents logistical issues for NER tool providers. These providers must not only develop the tool itself but must also create the Web service, make it available for use, and provide the necessary facilities and support for processing on an ongoing basis. It is well understood that these requirements may not be logistically feasible for all groups. However, in most cases the complexity imposed by making the tool available via Web services pales in comparison with the complexity imposed by developing the tool itself; the fact that so many effective Web service-based tools could be developed in conjunction with Track 3 in such a short period certainly validates this assertion. Moreover, modularizing and packaging tools for direct local third party integration presents its own set of significant logistical issues for the tool provider. The results of Track 3 strongly indicate that those groups that are interested in having their NER tools reach and benefit the broader research community, and have the wherewithal to develop and support their tools reliably within the context of a Web service framework, should strongly consider a Web service-based approach to dissemination.

From the client side, implementing a text-mining pipeline that integrates Web service-based calls to external NER tool providers certainly cedes some control of the pipeline to the Web service providers, and introduces the inherent risk of reliance on third parties. Issues such as Web service availability, support, robustness and response time variability must be carefully considered. Moreover, the viability of the organization to provide such services over the long term is also an important consideration. However, assuming reliable and effective NER Web services are available, and strong collaborative relationships can be developed with the Web service providers, the results of Track 3 clearly indicate that such an architecture can be simply and effectively implemented, has many other incumbent advantages and should be strongly considered by pipeline developers.

Looking ahead, CTD plans to pursue a Web service-based approach as a result of the Track 3 findings. More specifically, CTD plans to collaborate with the top performing teams in the individual NER categories, integrating their tools into the CTD text-mining pipeline. Testing will then be conducted to determine if the integration of these tools, either individually or in combination within NER categories, improves DRS scoring effectiveness and curation efficiency (through the identification and highlighting of genes, chemicals, diseases and action terms resident within the text being curated). CTD's use of BioC will be expanded, requiring added sophistication beyond that used for Track 3, including the addition of text/CTD controlled vocabulary translations, along with the specific locations of the terms within the text passages, and concept IDs. If testing is successful, these tools will be incorporated in earnest into the CTD text-mining pipeline.

In conclusion, the results of Track 3 underscore the extraordinary ability of Web services to abstract developers from the complexity of underlying computational systems, freeing them to purely focus on functional performance. In total, 44 platform-independent Web services, spanning four continents, encompassing four major NER categories, with varying levels of recall and precision, all using BioC as an interoperable common communications interchange framework, are now freely available for use; this is a significant accomplishment. One can only imagine how much more complicated and time-consuming it would have been for CTD to have attempted to test 44 independent NER tools by installing them locally, and in the absence of a common communications interchange framework; such an environment would likely have rendered testing, for all practical purposes, infeasible.

The fact that Track 3 has proven to be so successful brings with it the possibility that text-mining groups like CTD could mix-and-match NER functionality based solely on the expertise and performance of the NER provider, rather than on the characteristics of the respective tool's underlying technical architecture or geographic locale.

## Citing and linking to CTD

To cite CTD, please see http://ctdbase.org/about/publica tions/#citing. Currently, 51 external databases link to or present CTD data on their own Web sites. If you are interested in establishing links to CTD data, please notify us (http://ctdbase.org/help/contact.go) and follow these instructions: http://ctdbase.org/help/linking.jsp
